# Delivery of *Yersinia pestis* antigens via *Escherichia coli* outer membrane vesicles offered improved protection against plague

**DOI:** 10.1128/msphere.00330-24

**Published:** 2024-08-19

**Authors:** Zehui Tong, Xiangting Zhang, Xiao Guo, Gengshan Wu, Shiyang Cao, Yuan Zhang, Xiangze Meng, Tong Wang, Yiqian Wang, Yajun Song, Ruifu Yang, Zongmin Du

**Affiliations:** 1 State Key Laboratory of Pathogen and Biosecurity, Academy of Military Medical Sciences, Beijing, China; 2 Public Health School, Mudanjiang Medical University, Mudanjiang, China; University of Wyoming College of Agriculture Life Sciences and Natural Resources, Laramie, Wyoming, USA

**Keywords:** outer membrane vesicles, plague vaccine, *Yersinia pestis*, humoral immune response, immune protection

## Abstract

Outer membrane vesicles (OMVs) from Gram-negative bacteria can be used as a vaccine platform to deliver heterologous antigens. Here, the major protective antigens of *Yersinia pestis,* F1 and LcrV, were fused either with the leader sequence or the transmembrane domain of the outer membrane protein A (OmpA), resulting in chimeric proteins OmpA-ls-F1V and OmpA_46-159_-F1V, respectively. We show that OmpA-ls-F1V and OmpA_46-159_-F1V can be successfully delivered into the lumen and membrane of the OMVs of *Escherichia coli,* respectively. Mutation of *ompA* but not *tolR* in *E. coli* enhanced the delivery efficiency of OmpA-ls-F1V into OMVs. The OmpA-ls-F1V protein comprises up to 20% of the total protein in OMVs derived from the *ompA* mutant (OMV_dA_-ALS-F1V), a proportion significantly higher than the 1% observed for OmpA_46-159_-F1V in OMVs produced by an *ompA* mutant that expresses OmpA46-159-F1V, referred to as OMV_dA_-LATM5-F1V. Intramuscular (*i.m*.) immunization of mice with OMV_dA_-ALS-F1V induced significantly higher levels of serum anti-LcrV and anti-F1 IgG, and provided higher efficacy in protection against subcutaneous (*s.c.*) *Y. pestis* infection compared to OMV_dA_-LATM5-F1V and the purified recombinant F1V (rF1V) protein adsorbed to aluminum hydroxide. The three-dose *i.m*. immunization with OMV_dA_-ALS-F1V, administered at 14-day intervals, provides complete protection to mice against *s.c.* infection with 130 LD_50_ of *Y. pestis* 201 and conferred 80% against intranasal (*i.n*.) challenge with 11.4 LD_50_ of *Y. pestis* 201. Taken together, our findings indicate that the engineered OMVs containing F1V fused with the leader sequence of OmpA provide significantly higher protection than rF1V against both *s.c*. and *i.n*. infection of *Y. pestis* and more balanced Th1/Th2 responses.

**IMPORTANCE:**

The two major protective antigens of *Y. pestis*, LcrV and F1, have demonstrated the ability to elicit systemic and local mucosal immune responses as subunit vaccines. However, these vaccines have failed to provide adequate protection against pneumonic plague in African green monkeys. Here, *Y. pestis* F1 and LcrV antigens were successfully incorporated into the lumen and the surface of the outer membrane vesicles (OMVs) of *E. coli* by fusion either with the leader sequence or the transmembrane domain of OmpA. We compared the humoral immune response elicited by these OMV formulations and their protective efficacy in mice against *Y. pestis*. Our results demonstrate that the plague OMV vaccine candidates can induce robust protective immunity against both *s.c*. and *i.n. Y. pestis* infections, surpassing the effectiveness of rF1V. In addition, immunization with OMVs generated a relatively balanced Th1/Th2 immune response compared to rF1V immunization. These findings underscore the potential of OMVs-based plague vaccines for further development.

## INTRODUCTION

Outer membrane vesicles (OMVs) secreted by Gram-negative bacteria contain a variety of bacterial components, including membrane-associated lipopolysaccharides (LPS), peptidoglycans, and outer membrane proteins, alongside various components loaded inside their lumen (1). Many of these components are pathogen-associated molecular patterns (PAMPs) that can bind to innate immune system pathogen recognition receptors (PRRs) such as Toll-like receptors (TLRs) and NOD-like receptors (NLRs), thereby triggering robust innate immune responses and facilitating adaptive immunity. OMVs are non-replicative nanoscale particles that facilitate the uptake and presentation of carried antigens by antigen-presenting cells (APCs) while lacking the ability to induce associated diseases (2, 3). These characteristics of OMVs hold promise for their extensive applications. Heterologous antigens can be expressed in engineered bacteria and loaded into OMVs while retaining their native conformation and activity (4, 5). By fusing antigens with different carrier proteins or peptides, their expression in the outer membrane or periplasm of bacteria enables targeted localization to the outer membrane or lumen of OMVs (6).

OmpA is one of the most abundant outer membrane proteins in *E. coli* (7). It comprises an N-terminal leader sequence, a β-barrel transmembrane structure, and a C-terminal globular domain in the periplasm (8). When fused with heterologous antigens, the OmpA leader sequence, composed of 21 amino acid residues, can direct the secretion of these antigens into the bacterial periplasm and facilitate their presentation within the lumen of OMVs (6, 9). Previous studies have demonstrated the successful incorporation of heterologous antigens fused to the OmpA leader sequence into the lumen of OMVs, maintaining their native conformation and activity (9). Braun’s lipoprotein (Lpp), a major outer membrane lipoprotein in Gram-negative bacteria, contains an N-terminal signal peptide, followed by a cysteine-containing lipobox (10, 11). Fusion of the signal peptide and first nine amino acids of Lpp with five transmembrane regions of OmpA allows for the presentation of foreign antigens on the surface of *E. coli* outer membrane and their subsequent exposure on the external side of OMVs (12).

Plague is a highly lethal and contagious zoonosis caused by *Y. pestis,* with three major plague pandemics claiming approximately 200 million lives throughout history (13). Although the incidence of plague has gradually been reduced to lower levels, there has been an increasing trend in plague incidence in the 21st century, leading the World Health Organization (WHO) to classify it as a re-emerging infectious disease (14). The two major protective antigens of *Y. pestis*, LcrV and F1, have demonstrated the ability to elicit systemic and local mucosal immune responses, both individually and in combination, as subunit vaccines (15). However, previous studies have reported limitations in vaccine efficacy, prompting exploration into alternative vaccine strategies, such as OMV-based formulations (16, 17). In this study, we aimed to construct a recombinant fusion protein (F1V) and present it within OMVs localized in the lumen or outer membrane. We compared the humoral immune response elicited by these OMV formulations, as well as their protective efficacy in mice. Our results demonstrate that the plague OMV vaccine candidates are capable of inducing robust protective immunity against both subcutaneous (*s.c*.) and intranasal (*i.n*.) *Y. pestis* infections and merit further development.

## RESULTS

### Presentation of F1V on *E. coli* OMVs

The F1 antigen of *Y. pestis*, comprising 170 amino acids with a 21-amino acid leader sequence, plays a crucial role in plague immunity (18). After being secreted extracellularly, the mature F1 antigen, consisting of 149 amino acids and possessing a 15-amino-acid N-terminal signal peptide, assembles into a polymeric complex and forms a capsule. The LcrV antigen, vital for the development of a subunit vaccine against plague, contains immunosuppressive residues within amino acids 270–301 (19). A mutant form of LcrV mutant, lacking this region, exhibits reduced immunosuppressive activity compared to the full-length LcrV antigen (20).

**Fig 1:**
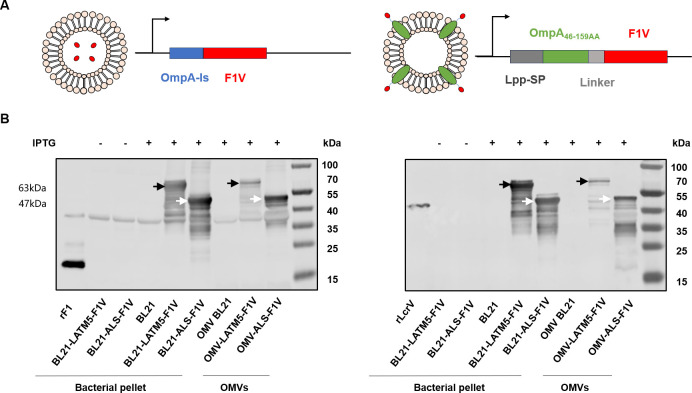
Expression of heterologous antigens in OMVs (**A**) Schematic representation illustrates the construction of OmpA-LS-F1V and Lpp-SP-OmpA_46-159_F1V fusion sequences. These fusion sequences consist of the OmpA leader sequence (blue), the Lpp signal sequence (gray), the OmpA_46-159_ fragment (green), a linker (light gray), and the F1V (red). (**B**) Western blot analysis to assess the expression of F1V and its localization on OMVs. Bacterial pellets and OMVs were prepared from BL21-ALS-F1V, BL21-LATM5-F1V, and BL21(DE3) strains with or without IPTG induction. Purified native F1 antigen and recombinant LcrV were used as controls. Immunoblotting was performed using mouse anti-F1(left) or anti-LcrV(right) monoclonal antibodies. Equal amounts of bacterial pellet samples (5 mg) and OMVs (10 µg) were subjected to SDS-PAGE and Western blot analysis. Black and white arrows indicated the chimeric F1V proteins with the expected size.

In this study, we truncated the first 12 amino acids of the F1 antigen and fused it with LcrV lacking the immunosuppressive region and subsequent sequences (rV270) (21) to generate the F1V fusion protein. This fusion protein was presented as a heterologous antigen on the OMVs of *E. coli* BL21(DE3) by two methods: one linked F1V downstream from the OmpA leader sequence, while the other connected F1V immediately downstream from, and in frame with Lpp signal sequence, the first nine N-terminal amino acids and the five transmembrane domains of OmpA in a sequential manner (Fig. 1A). The coding sequences for these fusion proteins were inserted into pET28a, resulting in the expression vectors pET28a-ALS-F1V and pET28a-LATM5-F1V. Subsequently, these vectors were transformed into BL21(DE3) to generate the strains BL21-ALS-F1V and BL21-LATM5-F1V. The results of immunoblot analysis indicated that proteins with molecular weights of 47 kDa and 63 kDa were detected in the bacterial pellet of BL21-ALS-F1V and BL21-LATM5-F1V, respectively, following IPTG induction. The OMVs were purified from the culture supernatant using tangential flow filtration (TFF) and ultracentrifugation. The resulting OMVs, named OMV-ALS-F1V and OMV-LATM5-F1V, exhibited successful incorporation of F1V, as confirmed by immunoblot analysis (Fig. 1B).

Interestingly, we observed that while expression levels of OmpA-ls-F1V and OmpA_46-159_-F1V in bacteria appeared comparable, OMV-ALS-F1V contained higher amounts of F1V protein than OMV-LATM5-F1V when they were equally loaded for analysis by SDS-PAGE and Western blot analysis (Fig. 1B). We speculate that this discrepancy could be attributed to the potential challenges in delivering the relatively large OmpA_46-159_-F1V protein (63 kDa) to the outer membrane.

### Enhancing antigen delivery efficiency in OMVs by mutation of *ompA* or *tolR*

Previous studies showed that mutation of *ompA* (22) or *tolR* (4, 23) can dramatically increase OMV production. Therefore, we proceeded to delete the *ompA* or *tolR* genes in *E. coli* BL21(DE3), resulting in the creation of BL21Δ*ompA* and BL21Δ*tolR* mutants. Subsequently, the pET28a-ALS-F1V and pET28a-LATM5-F1V plasmids were individually transformed into the *ompA* mutant, resulting in the generation of BL21_dA_-ALS-F1V and BL21_dA_-LATM5-F1V strains. Similarly, the transformation of these plasmids into the *tolR* mutant yielded the BL21_dR_-ALS-F1V and BL21_dR_-LATM5-F1V strains.

**Fig 2:**
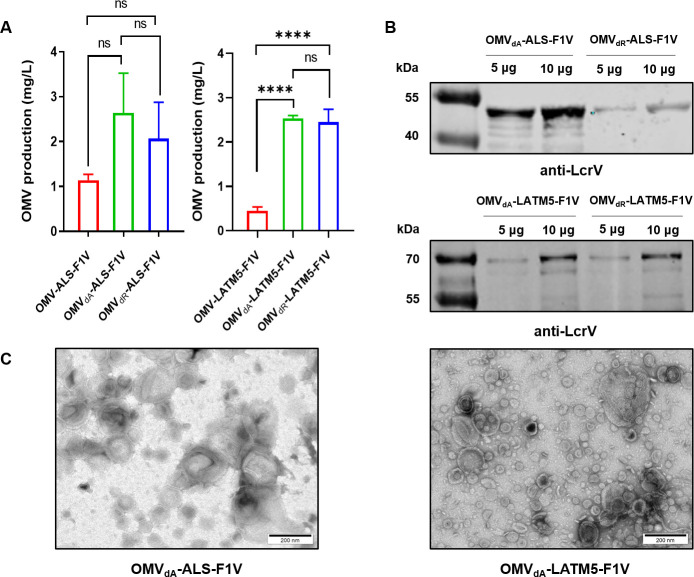
Analysis of OMVs from engineered *E. coli*. (**A**) Comparison analysis of OMV yields for OMV-ALS-F1V, OMV_dA_-ALS-F1V, and OMV_dR_-ALS-F1V (left), and OMV yields for OMV-LATM5-F1V, OMV_dA_-LATM5-F1V and OMV_dR_-LATM5-F1V (right). Statistical significance was determined using one-way ANOVA. *P < 0.05, **P < 0.01, ***P < 0.001, and ****P < 0.0001. (**B**) Comparison of F1V content in OMVs from *tolR* and *ompA* mutants by Western blot analysis. The efficiency of F1V presentation using the OmpA leader sequence is enhanced in BL21Δ*ompA*. (**C**) TEM images of OMVs purified from BL21_dA_-ALS-F1V and BL21_dA_-LATM5-F1V. Samples were stained using 2% phosphotungstic acid as described in the Materials and Methods and images were taken using a JEM1400 transmission electron microscope equipped with an XAROSA digital camera (scale bars, 200 nm).

OMVs secreted by these engineered strains were harvested, and the total protein concentration was quantified using the bicinchoninic acid assay (BCA) method. The protein concentration was used to represent the production of OMVs from the different strains. The results demonstrated that the OMV production from BL21_dA_-ALS-F1V and BL21_dR_-ALS-F1V were 2.64 mg/L and 2.07 mg/L, respectively, higher than the 1.14 mg/L yield from the BL21-ALS-F1V strain (Fig. 2A). Similarly, the OMV yields from BL21_dA_-LATM5-F1V and BL21_dR_-LATM5-F1V were 2.54 mg/L and 2.45 mg/L, respectively, whereas the OMV yield from BL21-LATM5-F1V was merely 0.45 mg/L (Fig. 2A). These findings indicated that deletion of the *ompA* or *tolR* gene effectively enhanced the production of OMVs in *E. coli*. In addition, no statistically significant difference in OMV yields was found between the *ompA* and *tolR* mutants (Fig. 2A).

Equal amounts of 10 µg of various OMVs were subjected to Western blot analysis. Interestingly, while the content of the F1V in OMV_dA_-LATM5-F1V and OMV_dR_-LATM5-F1V was essentially the same, the content of F1V in OMV_dA_-ALS-F1V was significantly higher than that in OMV_dR_-ALS-F1V (Fig. 2B). These findings suggest that incorporating heterologous antigens into OMVs via the OmpA leader sequence was facilitated by removing the native OmpA in *E. coli* while unaffected by the absence of TolR. Therefore, we employed the OMVs derived from the BL21Δ*ompA* strain in the following studies. Transmission electron microscopy (TEM) images (Fig. 2C; Fig. S1A) and nanoparticle tracking analysis (NTA) (Fig. S1B) revealed that different OMVs exhibited similar morphology and particle sizes. The average diameters of the OMVs secreted by *E. coli* BL21(DE3) and BL21Δ*ompA* were approximately 135.4 nm and 130.4 nm, respectively (Fig. S1B). Similar results for OMV_dA_-ALS-F1V and OMV_dA_-LATM5-F1V were obtained with average diameters of 121.8 nm and 130.2 nm, respectively (Fig. S1B).

### Determination of localization and quantity of F1V within OMVs

**Fig 3:**
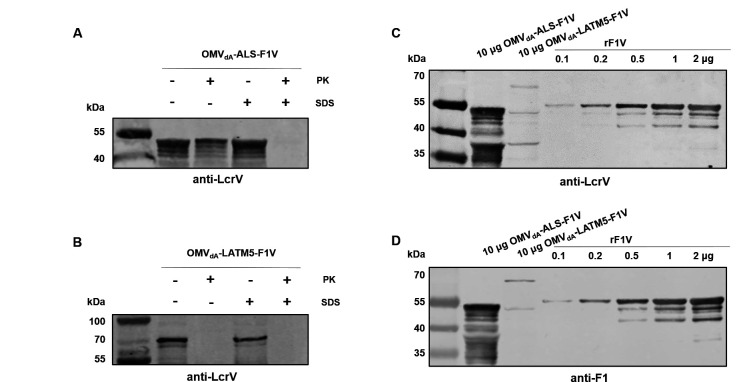
Analysis of localization and quantity of the F1V within OMVs. Purified OMVs, including OMV_dA_-ALS-F1V (**A**) or OMVdA-LATM5-F1V (**B**) were treated with proteinase K in the presence or absence of 1% SDS. Subsequently, samples were then subjected to SDS-PAGE and Western blot analysis using anti-LcrV antibody. The rF1V quantity within OMV_dA_-ALS-F1V (**C**) and OMV_dA_-LATM5-F1V(D) was determined by Western blot analysis.

To analyze the localization of F1V protein within two types of OMVs, OMV_dA_-ALS-F1V and OMV_dA_-LATM5-F1V were treated with proteinase K at 37°C for 15 minutes in the presence or absence of 1% SDS. Western blot analysis revealed that only a minor fraction of F1V within OMV_dA_-ALS-F1V was digested by proteinase K in the absence of 1% SDS, while complete digestion occurred in the presence of 1% SDS (Fig. 3A). This suggests that the F1V protein resided within the lumen of OMV_dA_-ALS-F1V. By contrast, in OMV_dA_-LATM5-F1V, F1V could be digested by proteinase K both with and without 1% SDS (Fig. 3B), indicating that F1V was exposed on the surface of OMV_dA_-LATM5-F1V.

For the quantification of F1V antigen within the OMVs, 10 µg of OMV_dA_-ALS-F1V and OMV_dA_-LATM5-F1V were analyzed by Western blot. Samples containing 0.1, 0.2, 0.5, 1, and 2 µg of the purified recombinant F1V (rF1V) protein were used as references. By comparing the band intensities of rF1V protein across different samples, it was estimated that approximately 2 µg of F1V was present in 10 µg of OMV_dA_-ALS-F1V, while only about 0.1 µg of F1V was detected in 10 µg of OMV_dA_-LATM5-F1V (Fig. 3C and D).

### Evaluation of the protective efficacy against *s.c.* and *i.n*. infection of *Y. pestis* in mice immunized with OMVs

Previous studies indicate that intramuscular (*i.m*.) immunization with OMVs may offer superior protection in mice compared to other frequently utilized immunization routes (4). Therefore, in this study, mice were *i.m*. immunized with two doses of different OMVs to assess their protective efficacy against the challenge of *Y. pestis* 201. Groups of mice (*n* = 20) received *i.m*. immunization with 60 µg of OMV_dA_-ALS-F1V, OMV_dA_-LATM5-F1V, OMVs extracted from BL21Δ*ompA* (OMV_dA_-NA), 12 µg of rF1V/Alhydrogel (rF1V) or phosphate-buffered saline (PBS) and were boosted at 21 days after the primary immunization (Fig. S2A). Given that every 10 µg of OMV_dA_-ALS-F1V contains roughly 2 µg of F1V, 12 µg F1V protein was utilized as a control for assessing the protective efficacy of the 60 µg of OMV_dA_-ALS-F1V. The immunized mice exhibited symptoms including ruffled hair, hunchback posture, and reduced appetite, and the weight losses post-vaccination were recorded (Fig. S2B). Mild swelling at the injection site was observed 1 week post-inoculation.

The vaccinated mice were challenged *s.c*. or *i.n*. with *Y. pestis* 201 on 42 days after the primary immunization and their survival was monitored for 14 days. As shown in Fig. S2, the OMV_dA_-ALS-F1V immunization afforded 80% protection against *s.c.* infection with 130 LD_50_ *Y. pestis* 201 (Fig. S2C), while rF1V immunization only provided 20% protection (Fig. S2C). No surviving mice were observed in the OMV_dA_-LATM5-F1V, OMV_dA_-NA, and PBS-immunized groups (Fig. S2C). After *i.n.* challenged with 11.4 LD_50_ *Y. pestis* 201, only 20% of the mice immunized with OMV_dA_-ALS-F1V or rF1V survived, with no mice in the OMV_dA_-LATM5-F1V, OMV_dA_-NA, and PBS-immunized groups surviving the same challenge (Fig. S2D). These results suggest that OMV_dA_-ALS-F1V is more effective than rF1V in preventing bubonic and pneumonic plague when an equivalent amount of F1V antigens is delivered via *i.m*. immunization.

**Fig 4:**
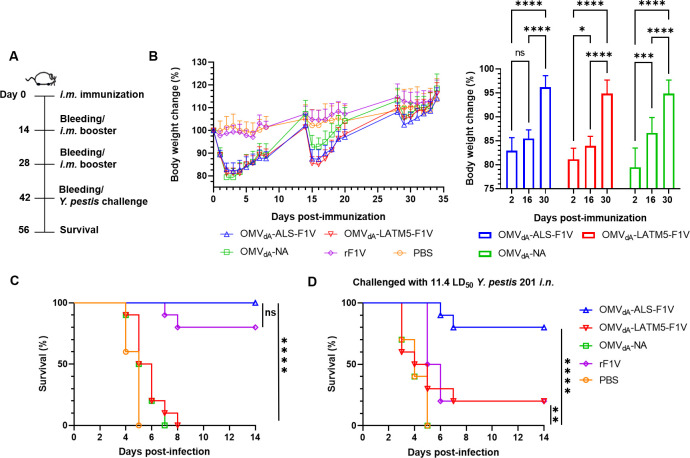
Efficacy of three-dose *i.m*. immunization of OMVs in protecting immunized mice against plague. (**A**) Immunization and challenge schemes were used for this experiment. BALB/c mice (*n* = 10, female) were *i.m*. immunized with 100 µL of PBS solutions containing 60 µg of OMV_dA_-ALS-F1V, OMV_dA_-LATM5-F1V, OMV_dA_-NA, or 12 µg of rF1V absorbed to the 100 µg Alhydrogel, while mice injected intramuscularly with PBS served as a negative control and then boosted on days 14 and 28 following the initial immunization. The vaccinated mice were then exposed to *s.c.* or *i.n*. challenge 14 days after the third immunization. (**B**) The body weight changes in mice were monitored following the three doses of *i.m*. immunization (left), along with a comparative analysis of the weight loss for each *i.m*. immunization event (right). Statistical significance was determined using one-way ANOVA with Tukey’s *post hoc* test, ns indicates no significance; *P < 0.05; **P < 0.01; ***P < 0.001; ****P < 0.0001. (**C**) On day 42 after the initial immunization, mice were *s.c.* challenged with 130 LD_50_ or (**D**) *i.n*. challenged with 11.4 LD_50_ of *Y. pestis* 201. Statistical significance was determined by the log-rank (Mantel–Cox) test. ns indicates no significance; *P < 0.05; **P < 0.01; ***P < 0.001; ****P < 0.0001.

Previous studies suggest that a three-dose immunization regimen could potentially enhance protection (24). Therefore, in the subsequent experiments, a three-dose immunization was implemented and the mice were administered a booster immunization on days 14 and 28 following the primary immunization (Fig. 4A). Remarkably, we observed that mice regained their body weight more rapidly after the third booster immunization and experienced significantly reduced adverse reactions. Specifically, the mean weight loss in the OMV_dA_-ALS-F1 V immunization group of mice was 17.09% 2 days after the initial immunization and 14.53% after the second booster, yet it significantly decreased to only 3.85% after the third immunization dose (Fig. 4B, right). The OMV_dA_-ALS-F1V immunization afforded complete protection against the *s.c.* challenge with 130 LD_50_ *Y. pestis* 201 (Fig. 4C), outperforming the rF1V-immunized group which exhibited a 70% survival rate (Fig. 4C). By contrast, none of the mice immunized with OMV_dA_-LATM5-F1V, OMV_dA_-NA, and PBS survived the same challenge (Fig. 4C). Following *i.n*. infection, the OMV_dA_-ALS-F1V immunization afforded 80% protection (Fig. 4D), while the OMV_dA_-LATM5-F1V and rF1V immunizations provided 20% protection, and neither the OMV_dA_-NA immunization nor PBS offer any protection against the same *i.n*. challenge (Fig. 4D). These results indicate that three doses of *i.m*. OMVs vaccination provide superior protection against pneumonic and bubonic plague compared to two doses. Subsequent experiments were conducted to evaluate the antibody titers in mice who received three doses of *i.m*. vaccination.

### Analysis of the humoral immune response elicited by different OMVs vaccination

**Fig 5:**
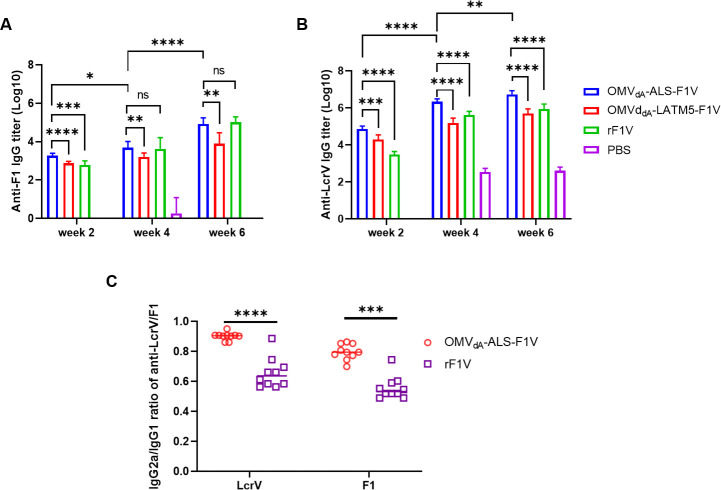
Antibody responses to LcrV and F1 in mice induced by *i.m*. immunization. BALB/c mice (*n* = 20, female) were *i.m*. immunized as described in Fig. 4. Sera were collected from the immunized mice (*n* = 10) at days 14, 28, and 42 after the initial immunization. Specific IgG against F1 and LcrV antigens was measured by ELISA. (A) Anti-F1 total IgG titers on weeks 2, 4, and 6 in different groups of the immunized mice. (B) Anti-LcrV total IgG titers on weeks 2, 4, and 6 in different groups of immunized mice. (C) Ratios of IgG2a/IgG1 for antibodies specific for the LcrV and F1 antigen on weeks 6. Data are presented as the mean ± SD. The significance of differences among groups was analyzed by two-way ANOVA with Tukey’s *post hoc* test (A, B) or paired *t*-test (C); ns indicates no significance; *P < 0.05; **P < 0.01; ***P < 0.001; ****P < 0.0001.

To assess the ability of various OMVs to elicit humoral immune responses in mice, serum samples were collected from mice who received a three-dose immunization, 14 days after each immunization. The serum samples were then analyzed using ELISA to measure the levels of anti-F1 and anti-LcrV-specific IgG titers. Our results revealed that following the initial immunization, the anti-F1 IgG titers in mice immunized with OMV_dA_-ALS-F1V were significantly higher compared to those in mice vaccinated with OMV_dA_-LATM5-F1V and rF1V (Fig. 5A). After the second and the third booster immunizations, the group immunized with OMV_dA_-ALS-F1V demonstrated titers comparable to the rF1V-vaccinated group and notably higher than the OMV_dA_-LATM5-F1V-immunized group (Fig. 5A). The specific anti-LcrV IgG titers in the mice immunized with OMV_dA_-ALS-F1V, OMV_dA_-LATM5-F1V, and rF1V reached a relatively high level following the primary immunization and gradually increased alongside the booster immunizations. Anti-LcrV IgG titers induced by the OMV_dA_-ALS-F1V consistently exceeded those induced by OMV_dA_-LATM5-F1V and rF1V with a significant difference (Fig. 5B). Taken together, these findings suggest that OMV_dA_-ALS-F1V is more effective in stimulating the humoral response in comparison to rF1V and OMV_dA_-LATM5-F1V.

In general, IgG1 is associated with a Th2-like response, while IgG2a is associated with a Th1 response (4, 25). Therefore, the ratio of IgG2a to IgG1 in the serum of immunized mice can reflect the relative strength of Th1 and Th2 response. The titers of IgG2a and IgG1 antibodies against LcrV and F1 in the serum of mice immunized with three doses of OMV_dA_-ALS-F1V and rF1V were measured, and the ratio of IgG2a/IgG1 was then calculated. The results indicated that the IgG2a/IgG1 ratios specific to LcrV and F1 in the OMV_dA_-ALS-F1V-immunized group were 0.90 and 0.79 (Fig. 5C), respectively, which were significantly higher than the ratios of 0.66 and 0.60 observed in the rF1V-immunized group (Fig. 5C). These results indicate that *i.m*. immunization with OMV_dA_-ALS-F1V generated a relatively balanced Th1/Th2 immune response compared to rF1V immunization.

## DISCUSSION

Heterologous antigens can be directly exposed on the surface of OMVs or encapsulated within the lumen of OMVs. Typically, it is believed that surface-exposed antigens are readily accessible to antigen-specific B cells, thereby eliciting robust humoral immune responses (6). However, Guido Grandi et al. have demonstrated that antigens can be incorporated into the OMVs lumen in their native conformation, also leading to the generation of high titers of protective antibodies in mice (9). To date, there is no definite conclusion regarding the impact of antigen localization on OMVs on immune efficacy. The incorporation of the two major protective antigens of *Y. pestis*, F1 and LcrV, into OMVs has been reported in bacteria including *Bacteroides thetaiotaomicron* (5) and *Y. pseudotuberculosis* (4, 26). In these studies, the expression of heterologous antigens within the bacteria was not consistently robust or was at a low level, and the localization of these antigens within OMVs has not been analyzed. For instance, after integrating the entire expression system of the F1 capsule of *Y. pestis* into the chromosome of the *Y. pseudotuberculosis*, the F1 antigen could not be detected in the bacterial pellet or OMVs by Western blot analysis (4).

Our previous studies have found that immunization of mice with rV270 in combination with native F1 antigen results in full protection of mice against a lethal dose of virulent *Y. pestis* (21). Therefore, in this study, after removing the leader sequence and signal peptide of the F1 coding sequence, coding sequences of F1_13-149_ and rV270 were fused with a linker to obtain the F1V fusion protein. We then devised two methods to present the F1V fusion protein on the outer membrane and lumen of OMVs, respectively, and compared their immunogenicity and protective efficacy against plague. Our study illustrates that OMVs generated by engineered *E. coli* can effectively deliver both major protective antigens against plague. Delivering F1V fusion protein by OmpA leader sequence into the lumen of OMV was very efficient, with F1V protein accounting for 20% of the total protein in the engineered OMVs, whereas delivering F1V by fusing with the transmembrane domain of OmpA to the surface of OMVs only constituted 1% total OMV protein. Both the OMV_dA_-ALS-F1V and OMV_dA_-LATM5-F1V-immunized groups produced a robust humoral immune response post*-i.m*. immunization, with the former demonstrating higher anti-F1 and anti-LcrV IgG titers in the serum (Fig. 5A and B). We speculate that this disparity might partially be attributed to the larger amounts of F1V contained in OMV_dA_-ALS-F1V compared to OMV_dA_-LATM5-F1V, resulting in enhanced protection efficiency against *Y. pestis* when mice were vaccinated with an equivalent dose of the two types of OMVs (Fig. 4C and D). These results underscore the significance of antigen delivery efficiency by OMVs for the immunogenicity of OMV vaccines. Upon comparing the protective efficacies of mice vaccinated with OMV_dA_-LATM5-F1V to those vaccinated with rF1V, we observed that while OMV_dA_-LATM5-F1V vaccination did not protect against *s.c*. challenge equivalent to rF1V, it did offer protection similar to rF1V against *i.n*. challenge.

Previous studies have indicated that OMV vaccines exhibit suboptimal efficacy when administered intranasally, whereas they are effective when administered intramuscularly or subcutaneously (4, 27). In this study, we validated the efficacy of OMV vaccines in mice via *i.m*. vaccination. Two doses of *i.m*. immunization with OMV_dA_-ALS-F1V provided 80% protection against *s.c.* challenges of *Y. pestis* 201. However, the protective efficacy against *i.n*. challenge was not satisfactory. Previous studies suggest that a three-dose immunization regimen can further enhance humoral response (24), prompting us to continue experiments with three-dose *i.m*. immunization using the same OMVs. The results indicated that while the IgG titers against LcrV exhibited only a slight increase post the third booster (Fig. 5B), the anti-F1 IgG titers underwent a substantial increase of approximately 16-fold from 1:6,400 to 1:102,400 (Fig. 5A). As a result, the protective efficacy of the OMVs administered in a three-dose regimen was significantly enhanced in protecting the vaccinated mice against the challenges of *Y. pestis* infection. The engineered *Y. pseudotuberculosis* strain-derived OMVs have demonstrated full protection against both *s.c*. and *i.n*. challenges with *Y. pestis*, despite the unsuccessful delivery of the F1 antigen through OMVs (4). The OMVs derived from *Y. pseudotuberculosis*, the progenitor of *Y. pestis*, may contain numerous antigens with protective immunity activity against *Y. pestis*, which we speculate to contribute greatly to the high level of protection efficacy observed.

It has been previously reported that both the F1 and LcrV subunit vaccines were able to elicit high IgG titers post-immunization (28). We observed that the specific anti-F1 IgG titers post-immunization with OMVs were notably lower than those for anti-LcrV. We speculate this discrepancy could be attributed to the ability of natural F1 to form linear polymerized structures (29), while the F1V fusion proteins exist exclusively as monomers, thus lacking the capacity to form the polymer structure and resulting in a diminished ability to induce humoral immunity. Our previous work showed that attenuated live vaccines could elicit high levels of anti-F1 IgG in mice but were much less effective in inducing specific anti-LcrV IgG antibodies (30). A future study combining OMVs vaccine with attenuated live vaccines through a sequential immunization approach holds promise for eliciting high antibody titers against both antigens simultaneously.

Antibody and cellular immune responses work synergistically to combat *Y. pestis* infection (31, 32). In line with the previous studies (4, 33), the IgG2a/IgG1 subtype ratio produced in OMVs-immunized mice in our study was consistently close to 1, surpassing that of the rF1V-immunized group. This suggests that OMVs can evoke a relatively balanced Th1/Th2 immune response (Fig. 5C), which is beneficial for stimulating both cellular and humoral immune responses to enhance vaccine efficacy. By contrast, attenuated live vaccines and subunit vaccines typically yield a lower IgG2a/IgG1 subtype ratio (30), highlighting the potential advantages of OMVs vaccines. Nonetheless, the mechanisms underlying the differences in humoral immune responses triggered by attenuated live vaccines, subunit vaccines, and OMVs vaccines remain incompletely elucidated and warrant further investigation.

PAMPs such as LPS in OMVs, although effective in activating innate immunity (34), are also the main cause of toxicity and adverse effects (35). Observations of the loss of body weight in mice after immunization with OMVs (Fig. 4B), along with other syndromes including ruffled hair, hunchback posture, and reduced appetite, underscore the notable reactogenicity associated with OMVs. The use of lipid A phosphatases such as LpxE (36) and LpxF (37) from *Francisella tularensis* offers a promising approach to mitigate the side effects associated with OMV vaccines. These enzymes catalyze lipid A into monophosphate lipid A (MPLA), which exhibits potent adjuvant activity while demonstrating significantly reduced toxicity compared to native lipid A (38, 39). Further studies focused on modifying the structure of lipid A hold promise for mitigating its toxicity.

## MATERIALS AND METHODS

### Bacterial strains and culture conditions

The bacterial strains used in this study are shown in Table S1. BL21(DE3) and DH5α *E. coli* strains were routinely grown in Luria-Bertani (LB) broth and LB agar medium at 37°C. All *E. coli* strains harboring pKD46 or pKD46-FnCpf1 were grown at 30°C in LB broth and LB agar to prevent plasmid loss. When required, kanamycin at 50 µg/mL, ampicillin at 100 µg/mL, and chloramphenicol at 25 µg/mL were added as antibiotic supplements to the culture medium. *Y. pestis* strains were routinely grown in LB broth medium at 26°C.

### Construction of plasmids and mutant strains

A sequence comprised of the coding sequence of 13–149 amino acids of F1 antigen and 1–270 amino acids of LcrV linked with a GSIEGR linker was fused to the OmpA leader sequence or in frame with the signal sequence and the first nine N-terminal amino acids of Lpp, and the amino acids 46–159 of the OmpA by synthesis directly between *Bam*H I and *Eco*R I in pET-28a (+), generating pET28a-ALS-F1V and pET28a-LATM5-F1V by Beijing Tsingke Biotech Co., Ltd. (40). The coding sequence for expression of rF1V was generated by a PCR using primer pairs rF1V-F and rF1V-R using pET28a-ALS-F1V as template. Then, the PCR product was inserted between the *BamH* I and *Sac* I to generate pET28a-rF1V, which was then transformed into BL21(DE3)-competent cells.

The *tolR* mutant of *E. coli* BL21(DE3) was constructed using the λRed recombinant system as previously reported (30, 41). Briefly, the kanamycin-resistant cassette from the plasmid pKD4 was amplified using primer sets *tolR*-P1/*tolR*-P2 (Table S2) and the PCR product was purified and electroporated into the competent cells *E. coli* BL21(DE3) harboring pKD46 to allow the replacement of the *tolR* with kanamycin-resistant cassette. After verification of the successful disruption of the *tolR* gene, the cassette was eliminated by introducing pCP20 (41), generating BL21Δ*tolR*. The strains were identified by PCR amplification with the primers *tolR*-F/*tolR*-R.

The *ompA* mutant of *E. coli* strain BL21(DE3) was constructed using CRISPR/Cas12a technology (42). The primers used are listed in Table S2. The up- and downstream fragments flanking the *ompA* gene were amplified using the primer pairs up-*ompA*-F/up-*ompA*-R and down-*ompA*-F/down-*ompA*-R, respectively. The PCR products were then mixed and used as templates for fusion PCR using up-*ompA*-F/down-*ompA*-R primer pairs to generate the *ompA* repair template. The product of fusion PCR was extracted from gels after electrophoresis analysis. The two complementary crRNA oligos targeting the *ompA* gene were annealed and cloned into *Bsa*I sites of pYC1000-eforRED to generate pYC1000-eforRED-*ompA*. pYC1000-eforRED-*ompA* was transformed into *E. coli* DH5α-competent cells, followed by plating on LB plates supplemented with chloramphenicol and incubation at 37°C overnight. After 18 hours, single colonies were picked and analyzed by DNA sequencing using the primer pYC1000-crRNA-cx. The mixtures of 400 ng pYC1000-eforRED-*ompA* and 700 ng *ompA* repair templet were electroporated into the competent cells of *E. coli* strain BL21(DE3) containing pKD46-FnCpf1. The bacterial suspensions were then cultured on LB agar plates containing ampicillin and chloramphenicol at 30°C. The colonies were identified by PCR amplification with the primers *ompA*-F/*ompA*-R. The plasmid pYC1000-eforRED-*ompA* was cured by growing the bacteria in LB with 7% sugar overnight and the plasmid pKD46-FnCpf1 was cured by culturing the bacteria in LB agar plates without antibiotics at 42°C for 48 hours and further identified by PCR amplification with the primers *sacB*-F/*sacB*-R or pKD46-cpf1-F/ pKD46-cpf1-R.

### OMV isolation

OMVs were isolated from *E. coli* strains as previously described with minor modifications (43). Briefly, bacterial strains were inoculated into 1 L LB broth and allowed to grow at 37°C with shaking at 200 rpm until reaching an optical density (OD) of approximately 0.6–0.8 at 600 nm. Protein expression was induced by isopropyl β-D-1-thiogalactopyranoside (IPTG) at a final concentration of 0.2 mM followed by incubation overnight at 16°C with shaking at 150 rpm. The culture supernatants were collected by centrifugation at 13,000 *× g* for 20 min at 4°C, passed through a 0.45-µm pore membrane (Corning), and concentrated with a 100-kDa filter using a Vivaflow 200 system (Sartorius). The concentrate was filtered through a 0.22-µm pore-size polyethersulfone filter (Millipore). OMVs were harvested by ultracentrifugation at 120,000 *× g* for 2 h at 4°C. The vesicle pellet was resuspended in 0.1× sterilized PBS (pH 7.4) and the ultracentrifugation step was repeated. The final vesicle pellet was resuspended in 0.1× sterilized PBS.

### Purification of the recombinant rF1V protein

The recombinant rF1V protein was purified as described in our previous study with minor modifications (44). Briefly, bacterial cells containing rF1V expression vector were cultured at 37°C until the OD_600_ reached about 0.6–0.8, and then induced by 0.2 mM of IPTG at 16°C for 12–14 h. Bacterial cells were harvested by centrifugation at 10,000 × *g* for 10 min and resuspended in lysis buffer containing 50 mM of NaH_2_PO_4_, 300 mM of NaCl, and 10 mM of imidazole (pH 7.5). After high-pressure freeze-fragmentation, the supernatant of the bacterial lysate was separated by centrifugation, and rF1V protein was purified with Ni-NTA Agarose (Qiagen, Hilden, Germany).

### Determination of protein concentrations in OMVs

Protein concentration in OMVs and the concentration of purified rF1V were quantified using a BCA protein analysis kit (Thermo Fisher Scientific). The F1V antigen presented in OMV preparations was detected by Western blotting.

### TEM analysis

The OMVs were analyzed by TEM as described previously with minor modifications (45). The vesicles were negatively stained with 2% phosphotungstic acid solution (, US) on a 400-mesh Formvar carbon-coated copper grid. The samples were viewed with a JEM1400 transmission electron microscope (JEOL, Peabody, MA) equipped with an XAROSA digital camera (EMSIS, Germany).

### Nanoparticle tracking analysis

OMVs’ particle size was measured using nanoparticle tracking analysis (NTA) at VivaCell Shanghai with Zetaview-PMX120-Z (Particle Metrix, Meerbusch, Germany) and corresponding software ZetaView (version 8.05.14 SP7)

### Western blotting analysis

Total bacterial proteins and OMVs suspensions were incubated in boiling water for 15 min after being suspended in an SDS-PAGE loading buffer. Samples were separated on 12% SDS-PAGE and then transferred onto a 0.45-µm polyvinylidene difluoride (PVDF) membrane. The PVDF membranes were blocked with 5% skimmed milk for 90 min at room temperature and incubated with anti-F1 or anti-LcrV monoclonal antibodies overnight at 4°C. After washing three times with TBST (0.05% Tween in TBS), the PVDF membranes were incubated at 37°C for 1 h in a 1:5,000 dilution of IRDye 800CW-conjugated goat-anti mouse secondary antibody. After washing three times with TBST, the immunoblotting results were imaged by an Odyssey SA imaging system (LI-COR Biosciences).

### Proteinase K treatment assay

OMV samples were incubated with proteinase K at a concentration of 100 µg/mL at 37°C for 15 min with or without the addition of 1% SDS (46). Proteinase K was then inactivated with 10 mM phenylmethylsulfonyl fluoride (PMSF) and the samples were analyzed by 12% SDS-PAGE followed by Western blotting as described above.

### Detection of antibody responses by ELISA

On days 14, 28, and 42 post-first immunization, sera were collected from immunized mice. The levels of IgG that recognize F1 or LcrV were evaluated by ELISA. Briefly, 96-well enzyme-linked plates (Dakewe Biotech Co., Ltd.) were coated overnight with 100 µL of 2 µg/mL of rF1 or 1 µg/mL of rLcrV. Then, 2% bovine serum albumin (BSA) was used to block the plates at 37°C for 2 h. Serum samples were serially diluted twofold and the antibody titers were measured as described previously (30, 47).

### Animal experiments

Seven- to eight-week-old female BALB/c mice were purchased from Vital River Laboratories (Beijing, China). Groups of mice (*n* = 20) were intramuscularly immunized with 100 µL of PBS solutions containing 60 µg of OMV_dA_-ALS-F1V, OMV_dA_-LATM5-F1V, OMV_dA_-NA, or 12 µg purified rF1V absorbed to the 100 µg Alhydrogel. Vaccination with PBS was used as a control. For the two-dose immunization experiment, a booster vaccination was administered 21 days after the initial vaccination. In the three-dose immunization experiment, booster vaccinations were given twice at 14-day intervals following the initial vaccination.

*Y. pestis* 201 was inoculated into LB and cultured at 26°C until the OD_620_ was approximately 1.0 for *s.c*. challenge experiment. For the *i.n*. challenge experiment, bacteria were inoculated into LB and incubated at 26°C until OD_620_ was approximately 0.6, followed by an additional 2-h incubation at 37°C. The bacterial cells were then collected by centrifugation at 4,500 rpm for 5 min, washed with PBS, and adjusted to an OD_620_ of approximately 1.0. and serially diluted to the appropriate concentration for use in the challenge. Our previous study showed that LD_50_ for the *s.c*. route of infection was determined to be 3.1 CFU, while for the *i.n*. route, it was found to be 439 CFU (30). At 42 days after the initial vaccination, the animals were anesthetized with iso pentobarbital (1.4 mg/each). Subsequently, they were either *i.n*. challenged with 10 µL suspensions of *Y. pestis* 201 (*n* = 10 for each group) or *s.c.* challenge at the groin with 100 µL suspensions of *Y. pestis* 201 in (*n* = 10 for each group) at the appropriate concentration. All infected animals were monitored over a 14-day period and the survival curves were plotted using GraphPad 9.0 software.

### Statistical analysis

Statistical analyses of comparisons of data among groups were performed with independent samples *t*-test or two-way ANOVA with Tukey’s *post hoc* tests. The log-rank (Mantel–Cox) test was used for survival analysis. All data were analyzed using GraphPad 9.0 software. The data are represented as the mean ± SD (ns, no significance; *P < 0.05; **P < 0.01; ***P < 0.001; ****P < 0.0001).

## SUPPLEMENTAL MATERIAL

Supplemental material msphere.00330-24-s0001.docx Fig. S1 and S2; Tables S1 and S2.

ASM does not own the copyrights to Supplemental Material that may be linked to, or accessed through, an article. The authors have granted ASM a non-exclusive, world-wide license to publish the Supplemental Material files. Please contact the corresponding author directly for reuse.
