# Supplementary material for: Delivery of Yersinia pestis antigens via Escherichia coli outer membrane vesicles offered improved protection against plague
Source: mSphere. 2024 Aug 19;9(9):e00330-24. doi: 10.1128/msphere.00330-24 (PMC11423571; doi:10.1128/msphere.00330-24)
Supplement: Supplemental material — Fig. S1 and S2; Tables S1 and S2. [file msphere.00330-24-s0001.docx]

**Supplemental materials**

**Supplemental Figures**


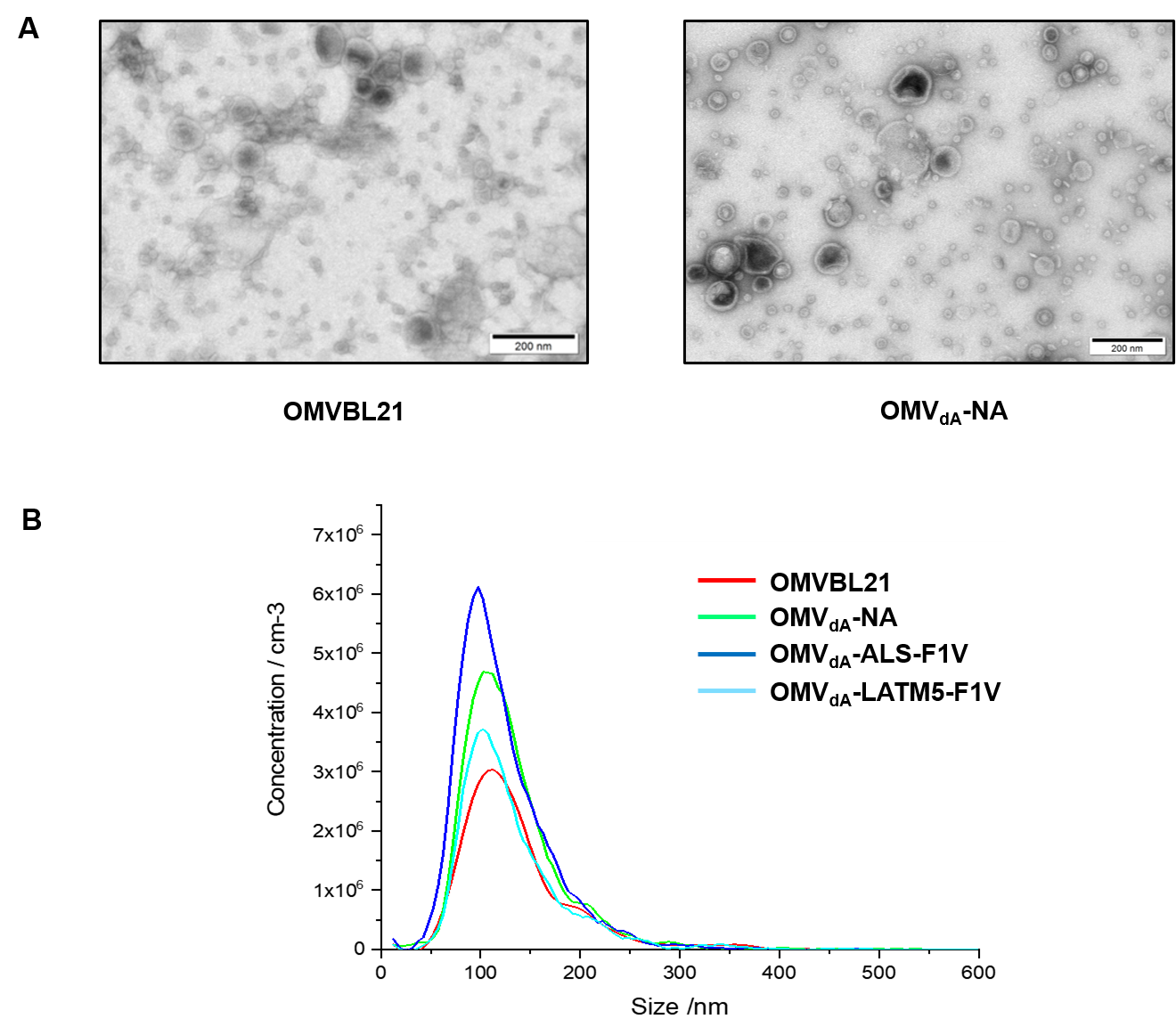


**Fig. S1. Analysis of OMVs derived from engineered *E. coli*.**

(A) TEM images of OMVs purified from BL21and BL21*ΔompA*. The samples were prepared by conventional staining with 2% phosphotungstic acid as described in the Materials and Methods. (Scale bars, 200 nm). (B) Size distribution of OMVs purified from the BL21，BL21*ΔompA*，BL21_dA_-ALS-F1V and BL21_dA_-LATM5-F1V strains.


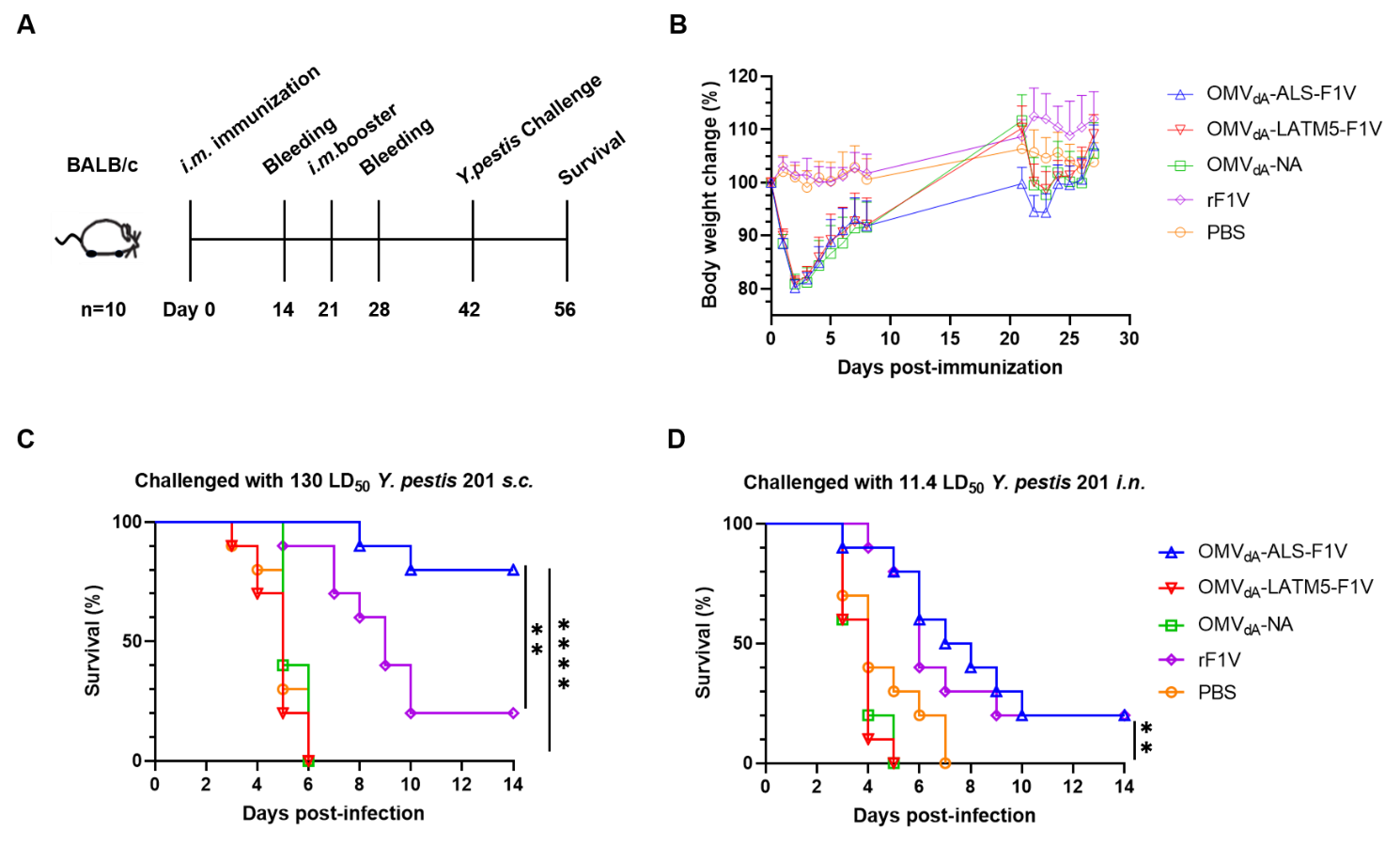


**Fig. S2. Evaluation of protective efficacy in mice following a two-dose *i.m.* immunization against plague.**

(A) Immunization and challenge schemes used for the animal experiment. BALB/c mice (n=10, female) were *i.m.* immunized with 100 μL of PBS solutions containing 60 μg of OMV_dA_-ALS-F1V, OMV_dA_-LATM5-F1V, OMV_dA_-NA, or 12 μg of F1V absorbed to the 100 μg Alhydrogel, while mice injected intramuscularly with PBS served as a negative control and then boosted on day 21 after the priming immunization. The vaccinated mice were then exposed to *s.c* or *i.n.* challenge 21 d after the second immunization. (B) Weight losses of mice after two-dose *i.m.* immunization. (C) On day 42 after the initial immunization, mice were *s.c* challenged with 130 LD_50_ or (D) *i.n.* challenged with 11.4 LD_50_ of *Y. pestis* 201. Statistical significance was determined by the log-rank (Mantel–Cox) test, ns indicates no significance; *P < 0.05; **P < 0.01; ***P < 0.001；****P < 0.0001.

**Supplemental Tables**

**Table S1. Strains and plasmids used in this study**

| **Strains or plasmids** | **Descriptions** | **Sources** |
| --- | --- | --- |
| **Strains** |  |  |
| *E. coli* DH5α | F-φ80 *lacZ*ΔM15 *Δ* (*lacZYA*-arg F) U169 *endA1 recA1 hsdR17* (*rk*^-^, *mk*^+^) *supE44* λ- *thi-1* *gyrA96 relA1 pho* | Laboratory collection |
| *E. coli* BL21(DE3) | *F–ompT hsdS (rB–mB–) dcm+Tetr galλ (DE3) endA Hte [argU proL Camr] [argU ileY leuW Strep/Specr]* | Laboratory collection |
| BL21-ALS-F1V | BL21(DE3) containing pET28a-ALS-F1V | This study |
| BL21-LATM5-F1V | BL21(DE3) containing pET28a-LATM5-F1V | This study |
| BL21*ΔompA* | BL21(DE3) *ompA* mutant | This study |
| BL21*ΔtolR* | BL21(DE3) *tolR* mutant | This study |
| BL21_dA_-ALS-F1V | *ΔompA* containing pET28a-ALS-F1V | This study |
| BL21_dA_-LATM5-F1V | *ΔtolR* containing pET28a -LATM5-F1V | This study |
| BL21_dR_-ALS-F1V | BL21*ΔompA* containing pET28a-ALS-F1V | This study |
| BL21_dR_-LATM5-F1V | BL21*ΔtolR* containing pET28a-LATM5-F1V | This study |
| *Y. pestis* 201 | Wild-type *Y. pestis* strain, avirulent to humans, highly virulent to mice | Laboratory collection |
| **Plasmids** |  |  |
| pKD46 | Temperature-sensitive plasmid expressing λRed recombinase under the control of arabinose; Ap^R^ | Laboratory collection |
| pKD4 | Template plasmid carrying antibiotic resistance genes that are flanked by FRT sites. | Laboratory collection |
| pCP20 | An Ap^R^ and Cm^R^ plasmid that shows temperature-sensitive replication and thermal induction of FLP synthesis | Laboratory collection |
| pKD46-Cpf1 | *Cpf1* inserted in pKD46 | Laboratory collection |
| pYC1000-eforRED | A Cm^R^ plasmid with *SacB* and *eforRed* for inserting the sgDNA sequence targeting the template DNA | Laboratory collection |
| pYC1000-eforRED-ompA | Constitutive expression of sgRNA editing ompA template DNA. | This study |
| pET28a-ALS-F1V | pET28a (+) derivative with insertion of OmpA leader sequence- F1_13-149_V_270_; Kan' | This study |
| pET28a-LATM5-F1V | pET28a (+) derivative with insertion of Lpp signal sequence-(1-9aa)-OmpA(46-159aa)- F1_13-149_V_270_; Kan^R^ | This study |
| pET28a-rF1V | pET28a (+) derivative with insertion of F1_13-149_V_270_; Kan^R^ |  |

**Table S2. Primers used in this study**

| **Name** | **Sequence** (**5’-3’**) | |
| --- | --- | --- |
| *tolR*-P1 | GATTCTGCACCGCCAGGCGTTTACCGTTAGCGAGAGCAACAAGGGGTAAGCC TGTGTAGGCTGGAGCTGCTTC | |
| *tolR*-P2 | CCAAAAACTGTTCGCCTGTTACCCGCTCTCTTTCAAGCAAGGGAAACGCAGATGT CATATGAATATCCTCCTTA | |
| *tolR*-F | CGCAAAAAGTTCTCGTCTGGTAGAAA | |
| *tolR*-R | TCCAACCGCTTTCAAGATT | |
| up-*ompA*-F | TTGACTGAAGAAGAGCATGCTGAAC | |
| up-*ompA*-R | CGAGAACTTTTTGCGCCTCGTTATCATC | |
| down-*ompA*-F | CGCAAAAAGTTCTCGTCTGGTAGAAAAACC | |
| down-*ompA*-R | AAAGCGGTTGGAAATGGAAGTATCTG | |
| crRNA-*ompA*-F | TAGATCCGGTCTTCGCTGGCGGTGTTGAG | |
| crRNA-*ompA*-R | AGACCTCAACACCGCCAGCGAAGACCGGA | |
| *ompA*-F | GCTTTACGCACGGGCAATTA | |
| *ompA*-R | TTACACGGTAACATTGAGATCGCTA | |
| rF1V-F | GTGGACAGCAAATGGGTCGCGGATCCATGCTTGTTGAACCAGCCCG | |
| rF1V-R | CTTGTCGACGGAGCTCGAATTCTTAGTGGTGGTGGTGGTGGTGGGC | |
| pCP20-F | TTGATGCGCTGGCAGTGTTC | |
| pCP20-R | GAGCTTTGTTGTAGGTGGACC | |
| pKD46-F | CTAAAAAAATCACTCACCCAG | |
| pKD46-R | TAATCAATCTACTCCCAAAGC | |
| pYC1000-crRNA-cx | TTCAGAGCAAGAGATTACGC |  |
| *sacB*-F | ACTTTTGGCTCGAGCATTCAAATATGTATCCGCTC |  |
| *sacB*-R | CGTTAAATAGCCGCTTATCATATGCACAGATGAAAACGG |  |
| pKD46-cpf1-F | CTAAAAAAATCACTCACCCAG |  |
| pKD46-cpf1-R | TAATCAATCTACTCCCAAAGC |  |

Note: Capital letters underlined indicate homologous DNA sequences used for gene mutation or plasmid construction via recombination cloning.
